# Photoreduction of Terrigenous Fe‐Humic Substances Leads to Bioavailable Iron in Oceans

**DOI:** 10.1002/anie.201600852

**Published:** 2016-04-21

**Authors:** Amir Blazevic, Ewelina Orlowska, Wolfgang Kandioller, Franz Jirsa, Bernhard K. Keppler, Myrvete Tafili‐Kryeziu, Wolfgang Linert, Rudolf F. Krachler, Regina Krachler, Annette Rompel

**Affiliations:** ^1^ Institut für Biophysikalische ChemieFakultät für ChemieUniversität WienAlthanstraße 141090ViennaAustria; ^2^ Institute of Inorganic ChemistryFaculty of ChemistryUniversity of ViennaWähringer Straße 421090ViennaAustria; ^3^ Institute of Inorganic ChemistryFaculty of ChemistryUniversity of ViennaAlthanstraße 141090ViennaAustria; ^4^ Institut für Angewandte SynthesechemieTechnische Universität WienGetreidemarkt 9/163-AC1060ViennaAustria

**Keywords:** complexed iron, dissolved organic matter, iron chelator agents, Fe K-edge, natural organic matter

## Abstract

Humic substances (HS) are important iron chelators responsible for the transport of iron from freshwater systems to the open sea, where iron is essential for marine organisms. Evidence suggests that iron complexed to HS comprises the bulk of the iron ligand pool in near‐coastal waters and shelf seas. River‐derived HS have been investigated to study their transport to, and dwell in oceanic waters. A library of iron model compounds and river‐derived Fe‐HS samples were probed in a combined X‐ray absorption spectroscopy (XAS) and valence‐to‐core X‐ray emission spectroscopy (VtC‐XES) study at the Fe K‐edge. The analyses performed revealed that iron complexation in HS samples is only dependent on oxygen‐containing HS functional groups, such as carboxyl and phenol. The photoreduction mechanism of Fe^III^‐HS in oceanic conditions into bioavailable aquatic Fe^II^ forms, highlights the importance of river‐derived HS as an iron source for marine organisms. Consequently, such mechanisms are a vital component of the upper‐ocean iron biogeochemistry cycle.

Iron is a micronutrient in oceans that directly contributes to sustaining life in marine systems and consequently it affects the global carbon cycle and the climate.^1^ The majority of the iron ligand pool in coastal and deepwaters (more than 99 %)^2^ is coordinated to organic scaffolds,^3^ which allows iron to exist in significantly higher concentrations (0.2 nm)^4^ than that permitted by the solubility limit of inorganic iron in water (<0.01 nm).^5^ In near coastal waters and shelf seas, the organic ligands primarily consist of aquatic humic substances (HS),^6^ making them highly important natural iron chelators and regulators of iron bioavailability.^7^ HS are predominantly composed of polyphenols and benzoic/carboxylic acids,^8^ but the structural association between iron and HS is poorly understood.^9^ It was shown recently that HS of terrestrial origin are a major class of iron chelator ligands, responsible for transportation of iron from the surroundings to the open sea.^10^ The biological uptake of iron from HS complexes in oceans has been proposed to occur through ligand substitution with siderophores,^6^ for which the exact mechanistic steps are still under investigation. Photochemical reactions result in reduction of Fe^III^ to Fe^II^ and have been reported for Fe^III^‐binding ligands of autochthonous marine origin like siderophores or freshly produced marine HS,^11^ but have not yet been reported for terrigenous HS in seawater.

X‐ray absorption near edge structure (XANES) and extended X‐ray absorption fine structure (EXAFS) techniques have previously been used to investigate Fe‐HS complexes in lakes and soils.^12^ However, no investigations describe Fe‐HS complexes during transport from terrestrial surroundings, via rivers, to oceans. HS representative of different geographical regions in Europe (TM=Tannermoor Brook, CB=Craggie Burn, and NR=Nordic reservoir; Table 1) and North America (SW=Suwannee River; Table 1) have been investigated in a combined X‐ray absorption spectroscopy (XAS) and valence‐to‐core X‐ray emission spectroscopy (VtC‐XES) study at the Fe K‐edge. The HS samples were investigated in freshwater and open ocean conditions after shaking in synthetic seawater,^13^ simulating estuarine zone mixing. In different aquatic environments, the set of HS samples address important aspects of the immediate Fe‐HS surroundings during transport to oceans, including stability and lifetime in open ocean conditions. The study also shows that photoredox cycling of Fe‐HS may lead to elevated steady‐state concentrations of bioavailable dissolved inorganic Fe^II^ species in open ocean conditions. For the first time, VtC‐XES^14^ experiments have been applied to HS chelating a transition metal, allowing assignment of the first shell ligands.^15^ The combined findings reported herein, strongly imply that river‐derived HS is an important iron source in the oceanic euphotic zone that allows for biological iron acquisition.

**Table 1:** A summary of the investigated HS samples. All samples, except CB0 and SW std., were in the liquid phase.

Sample details				Concentration (mg L^−1^)					
Origin	Code	Size fraction [kDa]	pH	[Fe]	DOC^[a]^	[N]	(F)/(O)^[b]^	Edge energy [eV]^[c]^	First shell coord.^[d]^			
Tannermoor	TM^[e]^	n/a	2.2	50.2	3160	63.7	O	7125.3	Fe^III^O_6_
Craggie Burn	CB^[f]^	n/a	7.7	18.9	1640	34.4	O	7125.1	Fe^III^O_6_
Suwannee River	SW^[g]^	n/a	7.6	3.5	115	2.4	O	7125.0	Fe^III^O_6_
Nordic reservoir	NR^[g]^	n/a	5.1	31	1150	23.8	O	7125.1	Fe^III^O_6_
Craggie Burn peak 0	CB0 13^[h]^	3.3	n/a	2.5^[l]^	50.2^[l]^	1.1^[l]^	O	7125.2	Fe^III^O_6_
Craggie Burn peak 0	CB0 14^[h]^	3.3	n/a	2.5^[l]^	50.2^[l]^	1.1^[l]^	O	7125.2	Fe^III^O_6_
Nordic reservoir 3/7	NR 3/7^[i]^	n/a	3.0	29	1150	23.8	O	7124.8	Fe^III^O_6_/Fe^II^O_6_ (90/10)
Nordic reservoir 3/21	NR 3/21^[i]^	n/a	3.0	29	1150	23.8	O	7123.2	Fe^III^O_6_/Fe^II^O_6_ (35/65)
Nordic reservoir 5/21	NR 5/21^[i]^	n/a	5.0	29	1150	23.8	O	7123.9	Fe^III^O_6_/Fe^II^O_6_ (65/35)
Tannermoor natural	TM nat.^[j]^	n/a	3.6	40	403	8.1	F	7125.3	Fe^III^O_6_
Suwannee standard	SW std.^[k]^	n/a	n/a	0.24^[l]^	52.5^[l]^	1.1^[l]^	F	7125.0	Fe^III^O_6_

[a] Dissolved organic carbon (DOC). [b] Freshwater samples (F) were investigated as collected in their natural environment, or as received from the International Humic Substances Society (IHSS). Ocean water samples (O) were subjected to estuarine mixing zone experiments by shaking portions (10 mg) of the solid NOM (aquatic natural organic material) with synthetic seawater^13^ (10 mL) for 30 minutes, followed by storage in a refrigerator at 4 °C for at least one week. All samples were kept in the dark at all times after sample collection, except for NR and CB0 13, which were subjects for iron photoreduction experiments. [c] Edge energy is based on the first maximum in the first derivative (Figure 1). [d] The percentages of NR reduction from Fe^III^O_6_ to Fe^II^O_6_ is based on LCF fitting (Supporting Information, Table S3). [e] TM collected in Upper Austria (geographic coordinates: N 48°30′ E 14°52′). [f] CB collected in North Scotland (geographic coordinates: N 58°26′ W 3°54′). [g] Environmental reference material, SW NOM from Florida, USA (RO isolation, 1R101N, from the IHSS), and NR NOM from Norway (RO isolation, 1R108N, from the IHSS) were used. [h] CB0 is the highest molecular weight size fraction of CB, which is explained in detail elsewhere.^16^ Samples CB0 13 and CB0 14 were prepared identically but collected one year apart. [i] The NR samples were exposed to natural daily sunlight for different time durations: NR 3/7 (pH 3, 7 days), NR 5/21 (pH 5, 21 days), and NR 3/21 (pH 3, 21 days). [j] Sample TM nat. was investigated as collected from the Tannermoor brook, without any shaking in synthetic seawater. [k] SW std. was investigated as received from the IHSS (RO isolation, 1R101N). [l] Solid samples (wt %).

The XANES spectra of 15 iron‐containing compounds (**1**–**15**) containing Fe^II^ and Fe^III^ oxidation states, and with different ligand scaffolds (Supporting Information, Figure S1) were investigated as references. The model compounds reflected the possible oxidation states and coordination modes that might be contained within the aquatic HS samples. The XANES spectra and the first derivative of the model compounds are shown in Figure S2 (Supporting Information), while the edge energies based on the first maximum in the first derivative are tabulated in Table S1 (Supporting Information). The edge energies of the model compounds span a range of about 5 eV, with increasing edge energy upon increasing oxidation state and electronegativity of the first shell atoms. Figure S3 (Supporting Information) shows the normalized XANES spectra and the first derivative of HS samples (Table 1) in both freshwater conditions (TM nat. and SW std.) and open ocean conditions (CB, CB0 13, CB0 14, NR, SW, and TM). The edge positions of the natural HS samples (that is, samples unexposed to light after sample collection) are found at 7125.1±0.2 eV. The shapes of the edges are closely comparable in all HS samples, indicating the same local coordination around the iron center in each of the samples. The extent of similarity is in agreement with previous investigations in terms of iron concentrations and size fractions present.10a, 16 The only observed difference is a pre‐edge peak with a slightly higher intensity in TM nat. compared to the remaining HS samples (Supporting Information, Figure S3) indicating a higher degree of non‐centrosymmetric coordination.

In Figure 1 the calculated coordination charges versus the experimentally determined Fe K‐edge positions are shown for model compounds **1**‐**15**. A straight line was regressed with a coefficient of determination *R* ^2^=0.95, demonstrating a linear correlation between the coordination charge and the edge positions.^17^ Electronegativity values determined from Allred/Rochow^18^ tables were used in the calculations, as reported elsewhere^17^, ^19^ (see Supporting Information for methods and Table S6). Using the calculations as a basis for the assignment of the iron oxidation state and coordinated first shell ligands and geometry in the HS samples, it is proposed that the majority of the iron centers are Fe^III^ coordinated in octahedral configuration by six oxygen donor atoms. The iron centers are suggested to be mononuclear in all HS samples, based on the fact that the oxygen bridged dinuclear model compounds **7** and **8** (Fe^III^O_6_; Supporting Information, Figure S2) differ markedly in the XANES region compared to the HS samples.

**Figure 1:**
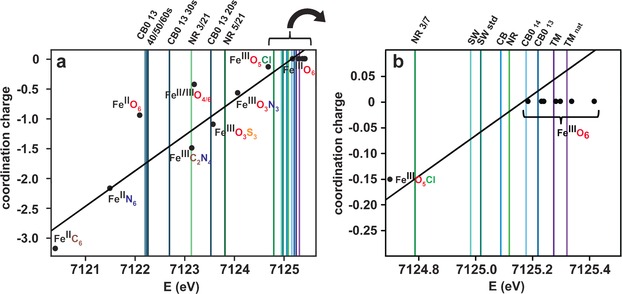
a) Calculated coordination charge *η*_AR_ according to the Allred‐Rochow^18^ scale in comparison to the observed edge energies in the XANES spectra of iron model compounds (dots) and HS samples (lines); b) enlarged section. The edge position over the first maximum in the first derivative was chosen as the point of reference for all model compounds and HS samples. The XANES spectra and corresponding first derivatives of iron model compounds are found in Figure S2 (Supporting Information), and that of the HS samples in Figure 2 a, b (NR and CB0 13) and Figure S3 (Supporting Information; SW, CB and TM samples).

All investigated HS samples were kept in the dark at all times after sample collection, except for two sets of samples containing NR or CB0 13. The NR (Figure 2 a) and CB0 13 10s (Figure 2 b) samples represent the initial iron oxidation state in the respective sets of samples, which was confirmed by comparing XANES spectra measured with low flux at two different beam lines, with no evidence of iron reduction (Supporting Information, Figure S4). In agreement with previous results,12a partial reduction of Fe^III^ to Fe^II^ was observed in NR samples exposed to diffuse natural sunlight on a daily basis (averaging 5 hours per day, at room temperature; Figure 2 a); namely, NR 3/7 (pH 3, 7 days of sunlight), NR 3/21 (pH 3, 21 days of sunlight), and NR 5/21 (pH 5, 21 days of sunlight).

**Figure 2:**
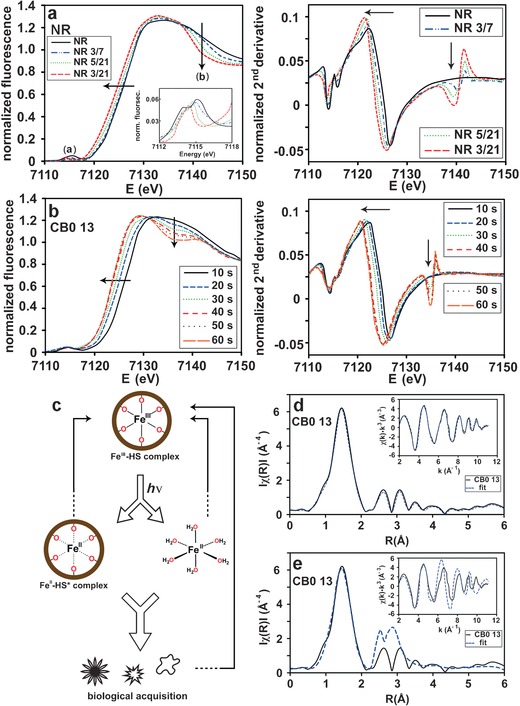
Normalized XANES region and the corresponding second derivative of a) NR and b) CB0 13 samples at different time intervals upon exposure to sunlight (a) and X‐rays (b), respectively. The inset in (a) shows an enlarged section of the pre‐edge region. Samples: NR 3/7 (pH 3, 7 days), NR 5/21 (pH 5, 21 days), and NR 3/21 (pH 3, 21 days). Smoothing has been applied to the spectra in (b) for improved visual appearance. c) Summary of photochemical iron cycling originating from river‐derived Fe‐HS complexes in the oceanic euphotic zone. d) and e) Two different fits of the same Fourier transform CB0 13 data. Both (d) and (e) show modeling of six oxygen/nitrogen contributions at 2.0 Å. Further away contributions from Fe‐C in (d) and Fe‐C/Fe‐Fe paths in (e) are modeled. The result from all fitted paths in the EXAFS is presented in Table S5 (Supporting Information). The inset in (d) and (e) show the k‐space data of CB0 13 with the corresponding fit. ARTEMIS^20^ and IFEFFIT^21^ were used to fit the experimental amplitudes and phases.

Besides a shift in the Fe K‐edges of iron in the NR samples, a small shift and sharpening of the pre‐edge peak (transformation from a centrosymmetric to a non‐centrosymmetric site^22^) is evident (feature (a) in Figure 2 a), as well as a loss in intensity of the shoulder at 7140 eV (feature (b) in Figure 2 a). The shift to a lower energy position, and decrease in height and area of the pre‐edge, marks a transformation toward a pre‐edge shape characteristic for Fe^II^O_6_ geometries in natural minerals.^22^ The reduction of Fe^III^ in CB0 13 by means of X‐rays (more than 10^10^ photons/second) is shown in Figure 2 b, where a shift in the Fe K‐edge to lower energies occurs the longer the sample is exposed. The average time needed to attain the same amount of intensity from natural solar irradiation at the ocean surface and 10 meters depth is in the range of hours (Supporting Information, Table S2). Photolysis experiments of Fe^III^ complexes with the marine siderophore aquachelin showed a similar timescale, with 70 % of Fe^III^ reduced into Fe^II^ after 4.5 hours.^11^ The averaged residence time of CB0 13 on the ocean surface would be 1.0 hour, and at 10 meters depth 5.3 hours (Supporting Information, Table S2).

Linear combination fitting was performed to obtain quantitative information on the most likely first shell coordination modes in the photoreduced NR and CB0 13 samples. The normalized spectra of HS were fitted (Supporting Information, Table S3) to model compounds **4** (Fe^III^O_6_), **5** (Fe^III^O_6_), and **13** (Fe^II^O_6_). The use of model compounds very similar to **4** and **5** were shown to give the best fitting results in an earlier study of HS.^23^ The fitting results of initial NR (48 % **4**, 52 % **5**) and CB0 13 10 s (42 % **4**, 58 % **5**) supports the previous assignment of Fe^III^O_6_ coordination. As reduction proceeds in the NR and CB0 13 samples, the amounts of **4** (Fe^III^O_6_) and **5** (Fe^III^O_6_) decrease. By comparison, **13** (Fe^II^O_6_) increases and reaches its highest proportions in NR 3/21 (64 %) and CB0 13 60s (61 %). The reduced Fe^II^ species may possess an Fe^II^O_6_ coordination environment, either as [Fe(H_2_O)_6_]^2+^ and/or as lower‐affinity Fe‐HS complexes available to biota, as exemplified in Figure 2 c.

The EXAFS of the Fe K‐edge model compounds **5** (Fe^III^O_6_), **6** (Fe^III^O_6_), **9** (Fe^III^O_3_N_3_), **10** (Fe^III^O_3_S_3_), **12** (Fe^III^C_2_N_4_), and **14** (Fe^II^N_6_) were measured and used to fit the study object, the high molecular weight fraction CB0 13. The model compounds contain different first shell neighbors and geometries that are likely to be present in CB0 13. The *k* ^3^‐weighted EXAFS spectra are shown in Figure S5 (Supporting Information) and all compounds were fitted close to crystallographic values using FEFF^24^ (Supporting Information, Table S4 and Figure S6). The oscillations in the fine structure of CB0 13 were extracted and fitted to paths in model compounds **1** (Fe^III^O_6_), **4** (Fe^III^O_6_), **5** (Fe^III^O_6_), and **9** (Fe^III^O_3_N_3_), leading to the satisfactory fit shown in Figure 2 d. Table S5 (Supporting Information) shows the results of the fits, which suggest back‐scattering of six oxygen/nitrogen atoms around the iron atom at 2.0 Å. This distance is typical for Fe^III^ in an octahedral configuration, and similar results have been described for Eliott Soil12a and Pahokee Peat HS12b in freshwater conditions. Thus, the presence of Fe^III^ in the HS samples is confirmed once more, as the bond distance for Fe^II^ is longer (2.10 Å).^25^ At roughly 2.95 and 3.92 Å contributions from Fe−C single scattering and Fe−O/N−C multiple scattering were observed, respectively. These Fe−C distances are present in complexes of Fe^III^ and small organic ligands,^23^, ^26^ and have been shown to exist in iron‐containing organic soils.12c Several types of functional groups containing oxygen atoms can be attributed to the Fe^III^ coordination environments, such as carboxyl, hydroxy and catecholic groups. Attempts to include Fe−Fe paths gave unsatisfactory fits with oscillations in k‐space that are out of phase with the experimental data (Figure 2 e).

VtC‐XES experiments were performed on model compounds **4** (Fe^III^O_6_), **9** (Fe^III^O_3_N_3_), and **10** (Fe^III^O_3_S_3_), as well as HS samples CB0 13, SW, and SW std., to allow resolution of the first shell composition and to distinguish between oxygen, nitrogen, and sulfur ligands. The spectral Kβ_2,5_ (metal 1s hole to ligand 2p (metal 4p)) and Kβ′′ (metal 1s hole to ligand 2s) transitions are given in Figure 3, which show distinct Kβ′′ lines that are well separated for oxygen/nitrogen and oxygen/sulfur contributions in **9** (Fe^III^O_3_N_3_) and **10** (Fe^III^O_3_S_3_), respectively.

**Figure 3:**
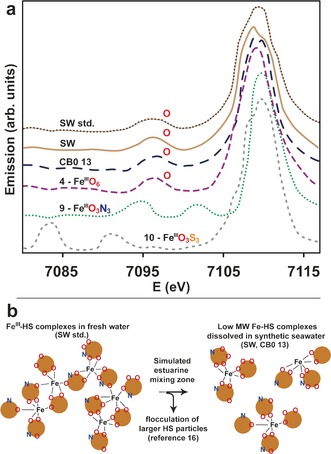
a) VtC‐XES spectrum of Kβ′′ satellite lines for **4**, **9**, **10**, CB0 13, SW and SW std. The spectra are plotted with an arbitrary vertical shift. b) Illustration of unchanged Fe^III^O_6_ first shell ligands between freshwater (SW std.) and open ocean condition (SW, CB0 13) samples, despite the potential to form complexes with nitrogen donor ligands. The ratio between oxygen and nitrogen donors is arbitrarily chosen since the oxygen:nitrogen ratios differ in all samples shown in Table 1.

Compound **4** (Fe^III^O_6_) shows only one Kβ′′ line, which is attributed to the six oxygen atoms in the first shell. SW, SW std., and CB0 13 are very similar to **4** (Fe^III^O_6_), unambiguously proving that iron is exclusively surrounded by oxygen atoms in the HS samples, which is an important complement to the XAS data. During transport from freshwaters to the ocean, while in the estuarine mixing zone, Fe‐HS complexes go through dissociation and reassociation of Fe‐HS of a lower molecular weight,^16^ as illustrated in Figure 3 b. Therefore, iron may potentially be coordinated by nitrogen‐containing functional groups as well. However, the preference for oxygen is strong as no nitrogen signal exists in either SW. std. (freshwater conditions), or SW and CB0 13 (open ocean conditions). This observation rules out the possibility of coordination to amines, despite the presence of nitrogen (1.1 wt % in solid samples; Table 1).

Fe^III^‐HS complexes are continuously released from terrestrial surroundings into freshwaters, and are subsequently transported to open oceans where they are photochemically reduced to crucial bioavailable iron species that are sequestered by phytoplankton. Light reactions in humic‐rich freshwaters are avoided to a large extent since they contain a significantly higher concentration of light absorbing substances compared to oceanic waters.^27^ Thus, oxidation state and coordination geometry remain unchanged for the majority of Fe‐HS complexes (octahedral Fe^III^O_6_; Figure 1) during transport from freshwaters to open oceans, despite their vastly varying geographical origins in the North Temperate Zone (CB, NR, SW, and TM; Table 1). Fe^III^‐HS in the high molecular weight fraction, CB0 13, form mononuclear complexes (Figure 2 d,e). Interestingly, no nitrogen atoms were identified in the first ligand shell of CB0 13, SW, and SW std. during the entire transport pathway (Figure 3 a) despite the possibility of forming new complexes with nitrogen donor ligands during estuarine zone mixing (Figure 3 b).^16^ This preference is unexpected since HS contain nitrogen (Table 1), which commonly serves as a donor atom for Fe^III^ species, but may be understood in terms of the stronger base characteristics of oxygen compared to nitrogen.^28^ Preferential Fe−O coordination is in line with iron coordination by marine siderophores, which has only seen complexation to oxygen‐containing functional groups thus far.^9^ Therefore, the river‐derived HS investigated herein reveal that nitrogen functional groups are of less importance as iron ligands in comparison with oxygen functional groups. Nitrogen‐containing species, such as amines, are consequently less significant as regulators of bioavailable iron in oceans.

Once NR and CB0 13 Fe^III^‐HS reach the ocean, they undergo photolytic intramolecular charge transfer reactions in which the iron is reduced to Fe^II^ and the ligand is oxidized (Figure 2 a,b). Reduction becomes more prominent with stronger light intensity and as pH decreases, leading to the formation of low‐affinity Fe^II^‐HS complexes and/or bioavailable Fe^II^, which may be sequestered by phytoplankton (Figure 2 c).^29^ The averaged residence time of Fe‐HS complexes at different oceanic depths fits well with oceanic iron cycling on a daily timescale. The Fe‐HS transport and photochemical cycling mechanisms described herein are likely to be fundamental features of iron release in comparison with other iron input methods in the oceanic euphotic zone.

## Supporting information

As a service to our authors and readers, this journal provides supporting information supplied by the authors. Such materials are peer reviewed and may be re‐organized for online delivery, but are not copy‐edited or typeset. Technical support issues arising from supporting information (other than missing files) should be addressed to the authors.

Supplementary
